# Supplementary material for: NMR-Based Characterization of Citrus Tacle Juice and Low-Level NMR and UV—Vis Data Fusion for Monitoring Its Fractions from Membrane-Based Operations
Source: Antioxidants (Basel). 2022 Dec 20;12(1):2. doi: 10.3390/antiox12010002 (PMC9854473; doi:10.3390/antiox12010002)
Supplement: Supplementary file 1 [file antioxidants-12-00002-s001.zip › antioxidants-2087435-supplementary.pdf]

NMR-Based Characterization of Citrus Tacle Juice and Low-Level NMR and UV—Vis Data Fusion for Monitoring Its Fractions from Membrane-Based Operations

Martina Gaglianò [1,2], Giuseppina De Luca [1,*], Carmela Conidi [2] and Alfredo Cassano [2,*]

[1] Department of Chemistry & Chemical Technologies, University of Calabria, Via P. Bucci, 87036 Rende, Italy

[2] Institute on Membrane Technology, ITM-CNR, 87036 Rende, Italy

[*] Correspondence: giuseppina.deluca@unical.it (G.D.L.); a.cassano@itm.cnr.it (A.C.); Tel.: +39-0984-493323 (G.D.L.); Tel.: +39-0984-492067 (A.C.)

Table S1: Metabolites identified in the 500 MHz ^{1}H Spectrum of the aqueous phase of Tacle juice at pH 3.41.

Compound	Assignment	^{1}H (ppm)	Multiplicity	^{13}C (ppm)
Amino acids				
Valine	γ-CH_3	0.98	d	
	γ'-CH_3	1.04	d	
Isoleucine	γ-CH_3	0.99		
	δ-CH_3	0.95		
Leucine	γ-CH_3	1.70		
	δ-CH_3	0.95		
Alanine	α-CH	3.79		
	β-CH_3	1.47	d	
Threonine	α-CH	3.96		
	γ-CH_3	1.22	d	
Arginine	β-CH_2	1.65;1.72	m	
	γ-CH_2	1.90	m	
	δ-CH_2	3.23		
GABA (γ-aminobutyric acid)	α-CH_2	2.46	t	
	β-CH_2	1.90	m	
	γ-CH_2	3.03	t	
Glutamine	α-CH	3.79		
	β-CH_2	2.13	m	
	γ-CH_2	2.53	m	
Proline	α-CH	4.13		
	β-CH	2.33	m	
	β'-CH	2.06	m	
	γ-CH_2	1.99	m	
	δ-CH	3.40		
	δ'-CH	3.33		
DMP (dimethylproline)	α-CH	4.07		
	β-CH	2.48	m	
	β'-CH	2.27	m	
	γ-CH_2	2.16	m	
	δ-CH	3.53		
	δ'-CH	3.69		
	N-CH_3	3.09	s	
	N-CH_3′	3.28	s	
Tyrosine	C2,6H ring	7.31	d	
	C3,5H ring	6.91	d	
Carbohydrates				
α-glucose	CH-1	5.22	d	94.83
	CH-2	3.53		73.80
	CH-3	3.70		
	CH-4	3.46		
	CH-5	3.83		
	CH_2-6,6	3.77;3.83		

β-glucose	CH-1	4.64		98.65
	CH-2	3.23	dd	76.93
	CH-3	3.48		78.54
	CH-4	3.38		72.38
	CH-5	3.46		78.62
	CH_2-6,6	3.73; 3.81		63.52
Sucrose	CH-1 (Glc)	5.40	d	94.83
	CH-2	3.57		73.80
	CH-3	3.75		75.55
	CH-4	3.47		71.95
	CH-5	3.85		75.07
	CH_2-6,6	3.80;3.82		62.88
	CH_2-1′ (Fru)	3.67		64.10
	CH-2′			104.2
	CH-3′	4.20		79.18
	CH-4′	4.04		76.93
	CH-5′	3.85		84.05
	CH_2-6′,6′	3.83		65.73
α-fructose	CH_2-1,1	3.58		65.70
	CH-3	4.09		78.18
	CH-4	4.09		78.18
	CH-5	3.82		83.36
	CH_2-6,6	3,68; 3.77		65.48
β-fructose	CH-3	3.78		70.34
	CH-4	3.88		72.45
	CH-5	3.99		77.20
	CH_2-6,6	3.69; 4.01		66.67
Organic Acids				
Lactic acid	α-CH	3.71		
	β-CH_3	1.22	d	
Succinic acid	α,β-CH_2	2.64	s	
Malic acid	α-CH	4.46		
	β-CH_2	2.72		
	β'-CH_2	2.86		
Citric acid	αγ-CH	2.78	d	46.6
	α'γ'-CH	2.91	d	46.6
Aspartic acid	α-CH	4.00		
	β-CH_2	2.71	dd	
	β'-CH_2	2.84	dd	
Formic acid	HCOOH	8.32	s	
Other compounds				
Ethanol	CH_3	1.17	t	
	CH	3.67		
Methanol	CH_3	3.32	s	
Phlorin	C4H ring	6.15	t	
	C2,6H ring	6.22	d	

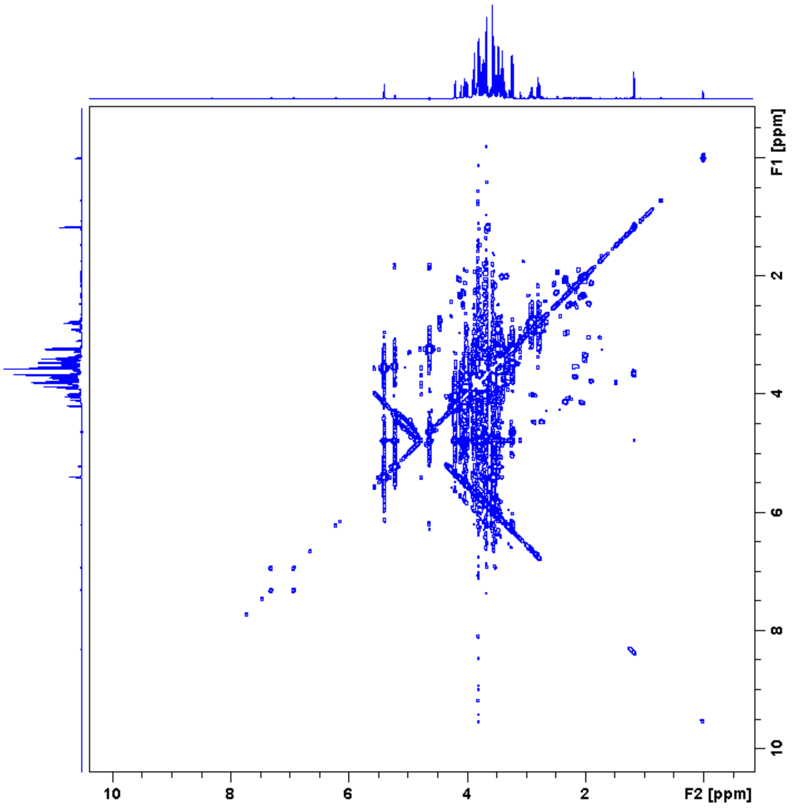

Figure S1: 2D ^{1}H COSY spectrum (Bruker pulse sequence: *cosygpprqf*) recorded on Tacle juice in D_2O (field strength of 11.74 T).

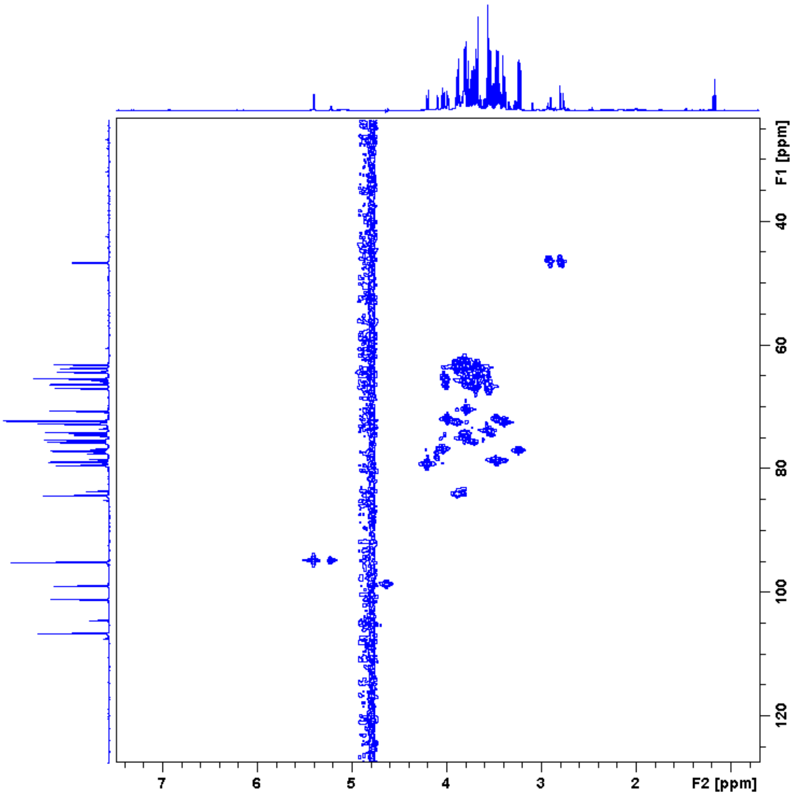

Figure S2: 2D ^{1}H-^{13}C HMQC spectrum (Bruker pulse sequence: *hmqcgpqf*) recorded on Tacle juice in D_2O (field strength of 11.74 T).
